# Combined effects of calcium sources and water restriction on fruit yield and quality of ‘Ataulfo’ and ‘Kent’ mangoes

**DOI:** 10.3389/fpls.2025.1622533

**Published:** 2025-08-28

**Authors:** Lucas Soares Rodrigues, Antônio Gustavo de Luna Souto, Ítalo Herbert Lucena Cavalcante, Lucas Henrique Maciel Carvalho, Jamiles Carvalho Gonçalves de Souza Henrique, Gilberto José Nogueira e Silva, Vespasiano Borges de Paiva Neto, Rejane Maria Nunes Mendonça, Hans Raj Gheyi, Geovani Soares de Lima, Rafael Oliveira Batista, Cícero Henrique de Sá, Wedson Aleff Oliveira da Silva, Victor Daniel de Lima Cunha, Abraão Targino de Sousa Neto

**Affiliations:** ^1^ Programa de Pós-Graduação em Agronomia, Universidade Federal da Paraíba, Areia, Paraíba, Brazil; ^2^ Programa de Pós-Graduação em Manejo de Solo e Água, Universidade Federal Rural do Semi-Árido, Mossoró, Rio Grande do Norte, Brazil; ^3^ Programa de Pós-Graduação em Agronomia – Produção Vegetal, Universidade Federal do Vale do São Francisco, Petrolina, Pernambuco, Brazil; ^4^ Programa de Pós-Graduação em Engenharia Agrícola, Universidade Federal de Campina Grande, Campina Grande, Paraíba, Brazil; ^5^ Departamento de Agronomia, Universidade Federal do Vale do São Francisco, Petrolina, Pernambuco, Brazil; ^6^ Programa de Pós-Graduação em Fitotecnia, Universidade Federal Rural do Semiárido, Campina Grande, Paraíba, Brazil

**Keywords:** Mangifera indica l., plant nutrition, pulp composition, calcium complexes, fruit postharvest

## Abstract

**Introduction:**

Mango is Brazil’s leading fruit export in terms of both volume and value. Competitiveness in the international market demands high-quality fruits free from physiological disorders, with calcium (Ca^2+^) nutrition and irrigation management being key factors in this context. Therefore, this study aimed to evaluate the effect of calcium sources and preharvest water restriction on yield and postharvest quality of ‘Ataulfo’ and ‘Kent’ mangoes grown in the São Francisco Valley, Brazil.

**Methods:**

Two experiments (‘Ataulfo’ and ‘Kent’ mangoes) were conducted in a randomized block design, arranged in a 2 × 4 factorial scheme (with and without water restriction; no Ca^2+^, calcium chloride - CaCl_2_, calcium complexed in organic acids - Ca-OA, and calcium complexed in amino acids -Ca-AA), with four replicates. Variables related to yield, physicochemical quality, mineral composition of the pulp, and cell wall-bound calcium were assessed.

**Results:**

The application of complexed calcium sources (Ca-OA and Ca-AA) improved fruit quality and fruit yield, with cultivar-specific responses. Ca-OA was moreeffective under full irrigation, whereas Ca-AA was more efficient under water stress, by decreasing the N:Ca ratio and increasing cell wall-bound calcium.

**Discussion:**

For ‘Ataulfo’, the use of Ca-AA combined with preharvest water deficit is recommended. For ‘Kent’, irrigation should be maintained, and Ca-AA should be used as the preferred calcium source. The combination of complexed calcium and irrigation management can optimize mango production and quality in semiarid regions.

## 1 Introduction

Mango (*Mangifera indica* L.) stands among Brazil’s most economically significant fruit crops, leading the country’s fresh fruit exports in 2023 (Brasil, Ministério da Agricultura e Pecuária, 2023). In 2024, the São Francisco Valley emerged as the dominant production hub, accounting for 93% of national mango exports, thus consolidating its strategic importance for both domestic supply and international trade (Brasil, Ministério da Agricultura e Pecuária, 2024).

Among the key quality attributes that determine export value, high accumulation of dry matter in the pulp and the absence of physiological disorders are particularly critical (Camara, 2017). Several studies have identified dry matter content at harvest, along with other traits, as a reliable indicator of fruit maturity and postharvest quality (Anderson et al., 2020). Quality of mango fruit is strongly influenced by nutritional management, particularly calcium (Ca^2+^) availability (Freitas and Mitcham, 2012; Tenreiro et al., 2023; Sena et al., 2024), and irrigation practices. As noted by Anderson et al. (2017), water deficits during early fruit development may limit fruit size, whereas deficits imposed during the final stages of development can enhance the concentration of storage reserves (carbohydrates).

Calcium is among the most vital nutrients for maintaining mango postharvest quality (Sena et al., 2024). Besides its essential roles in metabolic functions such as protein synthesis, nitrogen metabolism, enzymatic activation, and the transport of carbohydrates and amino acids (Hocking et al., 2016; Thor, 2019), calcium contributes significantly to fruit development and the prevention of physiological disorders (Assis et al., 2004; Haleema et al., 2024; Torres et al., 2024).

Despite its physiological importance and being one of the most absorbed nutrients in mango plants (Silva et al., 2021; Rezende et al., 2022), and the third most exported to the fruit (Maldonado-Celis et al., 2019) calcium exhibits limited phloem mobility, depending primarily on xylem transport (Epstein and Bloom, 2005; Marschner, 2023). When calcium supply is inadequate or unevenly distributed, localized deficiencies can occur, directly impairing fruit integrity and postharvest performance (Torres et al., 2024; Kabir and Díaz-Pérez, 2025).

Moreover, calcium ions function as pivotal secondary messengers in various plant signaling pathways (Thor, 2019). Environmental and endogenous stimuli trigger distinct cytosolic Ca²^+^ fluctuations, which are perceived by specific sensor proteins, ultimately leading to changes in cellular activities and the regulation of gene expression (Verna et al., 2022). Calcium ions act as crucial second messengers in plants, playing indispensable roles in growth, development, and responses to both biotic and abiotic stressors (Feng et al., 2023). Upon perceiving environmental cues such as pathogen attack, salinity stress, temperature fluctuations, or nutrient deficiency, plants exhibit rapid and transient increases in cytosolic calcium concentration ([Ca²^+^]cyt), forming specific patterns known as “Ca²^+^ signatures” (Lu et al., 2025).

These signatures are central to signal transduction and the activation of adaptive cellular responses, including gene expression, metabolic adjustments, and transport regulation (Shu et al., 2024). The generation and regulation of these signals involve specific calcium influx and efflux channels, whose mechanisms have been increasingly elucidated in recent years. However, excessive Ca²^+^ accumulation poses a risk of cytotoxicity, requiring tight control of calcium gradients within plant cells (Naz et al., 2024).

Water stress activates physiological responses such as stomatal closure, osmoprotectant accumulation (proline and soluble sugars), antioxidant defense, and hormonal adjustments, especially increased abscisic acid (ABA), that can favor fruit formation and improve quality traits such as sugar content and firmness. Moderate water deficit has been shown to enhance sugar accumulation and improve fruit taste without compromising shape, as observed in crops like tomato and apple (Lu et al., 2019). These adjustments are mediated by ABA-regulated signaling pathways and changes in cellular osmotic balance, which help orchestrate the plant’s adaptive responses under stress (Shu et al., 2024).

Given these limitations, new calcium fertilization strategies have been explored to improve uptake and translocation, particularly using complex formulations. Unlike conventional sources such as calcium chloride, limestone, or gypsum, formulations that include humic substances, seaweed extracts, chitin and its derivatives, antitranspirants, free amino acids or organics acids may increase calcium solubility, absorption, and effectiveness (Mengel and Kirkby, 2001; Du Jardin, 2015; Ram et al., 2024). These agents enhance calcium availability and may facilitate its movement to sink organs such as fruits.

Irrigation management also plays a pivotal role in fruit development and quality (Anderson et al., 2017), though findings remain variable (Rodrigues, 2023). For example, Castro Neto and Reinhardt (2003) observed that preharvest water deficit reduced dry matter accumulation in ‘Haden’ mangoes, a potentially adverse outcome. In contrast, studies with ‘Tommy Atkins’ mangoes (Santos et al., 2016a; 2016b) demonstrated that controlled water deficit during ripening did not reduce yield and improved water use efficiency. Anderson et al. (2017) further reported that suspending irrigation 2 to 4 weeks before harvesting increased dry matter accumulation without compromising fruit size or ripening uniformity.

The combined use of calcium sources and preharvest water restriction is based on the hypothesis that their interactions, whether synergistic or antagonistic, can affect physiological responses and fruit quality. While calcium reinforces structural and metabolic stability, moderate water stress may modulate defense mechanisms and enhance solute accumulation, potentially improving postharvest attributes.

Given this context, the present study aimed to evaluate the effects of calcium sources and preharvest water restriction on fruit yield and postharvest quality of ‘Ataulfo’ and ‘Kent’ mangoes grown in the São Francisco Valley, Brazil.

## 2 Materials and methods

### 2.1 Climate and soil characterization of the experimental areas

Two experiments with ‘Ataulfo’ and ‘Kent’ mango trees were conducted simultaneously at Casa Nova and Nogueira Farms, respectively, from September 2021 to October 2022. The Casa Nova farm, Casa Nova, Bahia, Brazil is georeferenced at coordinates 9°19’40.5”S and 41°07’55.9”W, at an elevation of 400 m above sea level, while the Nogueira Farm, Petrolina, Pernambuco, Brazil is located at 9°21’42.7”S and 40°38’01.4”W, at an elevation of 395 m above sea level.

**Figure 1:**
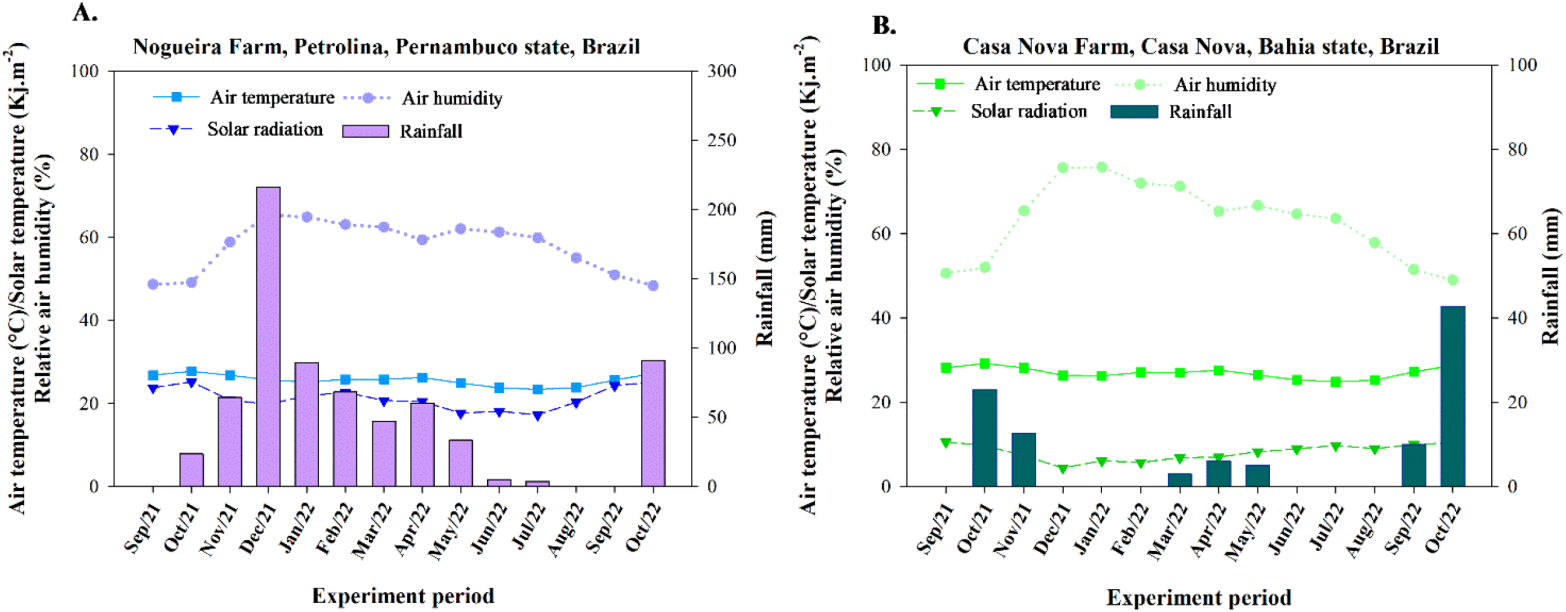
Average air temperature, solar radiation, relative humidity of the air, and rainfall recorded during the experiments. **(A)** Experiment conducted in Petrolina, at Nogueira Farm; **(B)** Experiment conducted in Casa Nova, at Casa Nova Farm.

The climate in the experimental region is classified as BSwh according to the Köppen climate classification system, corresponding to a hot semi-arid climate (Alvares et al., 2013). Throughout the experimental period, monthly data on rainfall, air temperature, and relative humidity of the air were recorded by automatic weather stations located near the experimental areas (**Figure 1**).

Prior to the establishment of the experiments, soil samples were collected from a depth of 0 to 30 cm and analyzed to determine the chemical properties for soil fertility in each experimental area (**Table 1**). Leaf samples were collected from the last mature vegetative flush and stored in paper bags. Leaf collection criteria followed the recommendations of Malavolta et al. (1997). After washing with distilled water, the leaves were placed in a paper bag for drying in a forced-air oven at 60°C until constant mass, ground in a stainless-steel knife mill (Wiley type) and stored in a hermetically sealed container. The samples were analyzed for N (g kg^-1^), P (g kg^-1^), K (g kg^-1^), Ca (g kg^-1^), Mg (g kg^-1^), S (g kg^-1^), B (mg kg^-1^), Fe (mg kg^-1^), Cu (mg kg^-1^), Mn (mg kg^-1^), and Zn (mg kg^-1^) according to the methodology proposed by Tedesco et al. (1995) and results obtained are presented in **Table 2**. Both soil and leaf analysis were realized by the Plant Soil Laboratories™.

**Table 1:** Chemical analysis of the soil in the experimental area prior to treatment application.

Cultivar	pH	O.M	P	K^+^	Na^+^	Ca^2+^	Mg^2+^	Al^3+^	(H^+^ + Al^3+^)	CEC	V	Sat. Ca
	H_2_O	g 100g^-1^	mg dm^-3^	^__________________^ cmol_c_ dm^-3_______________________^						%		%
Ataulfo	6.21	17.1	34.3	0.12	0.06	3.40	0.95	0.00	0.45	4.98	90.90	68.2
Kent	6.81	19.4	11	0.27	0.03	2.39	0.54	0.00	0.76	3.99	80.95	59.9

Extractors: P, K and Na: Resin (HCl + H_2_SO_4_); Ca, Mg and Al: KCl 1 M; CEC, Cation exchange capacity; V, Base saturation; Sat. Ca: Calcium saturation.

**Table 2:** Nutritional analysis of mango leaves prior to treatment application.

Cultivar	N	P	K^+^	Ca^2+^	Mg^2+^	S	B	Cu^2+^	Fe^2+^	Mn^2+^	Zn^2+^
^ ___________________________^ g kg^-1 _________________________^							^_______________________^ mg kg^-1 ________________________^				
Ataulfo	14.8	1.3	4.5	27.4	1.8	2.1	57.1	10.1	111.4	150.9	15.4
Kent	13.7	1.2	3.8	29.0	2.7	1.6	115.2	7.2	130.8	646.9	29.9

### 2.2 Experimental design and plant material

The experiments with ‘Ataulfo’ and ‘Kent’ mango trees were arranged in a randomized block design in a 2 × 4 factorial scheme, with four replications and three plants per plot. The factors consisted of water deficit imposed by irrigation suspension 15 days before harvest (with and without) and calcium sources [no calcium (control); CaCl_2_ – calcium chloride; Ca-OA – calcium complexed with organic acids; and Ca-AA – calcium complexed with amino acids). ‘Ataulfo’ mango has smaller fruits (180–250 g), with thin skin, creamy pulp, and high sugar content, with average yield between 10 and 19 t ha^-1^ (Flores et al., 2024); while ‘Kent’ mango has larger fruits (400–600 g), with thick skin, firm pulp, and low fiber content, with an average yield between 33 and 50 t ha^-1^ (Lobo et al., 2019). ‘Kent’ mango usually has higher yiled, and ‘Ataulfo’ mango stands out for its precocity and better adaptation to moderate water stress.

At the beginning of the experiment, ‘Ataulfo’ mango trees were five years old, spaced at 10 m × 10 m (population density = 100 plants ha⁻¹), and irrigated daily using a localized micro-sprinkler irrigation system with emitters for a flow of 60 L h⁻¹ at a working pressure of 0.2 MPa. The ‘Kent’ mango trees were four years old, spaced at 3.5 m × 2.0 m (1,428 plants ha⁻¹), and irrigated daily via a localized drip irrigation system using dual drip tape with four emitters per plant, with an individual flow of 2.4 L h⁻¹.

At the beginning of the experiment, calcium fertilization demands were performed to raise calcium saturation in the soil cation exchange capacity (CEC) to 75% for ‘Ataulfo’ and 70% for ‘Kent’ planned in two stages:

(i) During the pre-pruning phase, based on the soil’s chemical attributes, according to the base saturation balance method described by Albrecht (1975). In the area with ‘Ataulfo’ mango trees, calcium saturation was increased from 68.2% (**Table 1**) to 70%, and in the ‘Kent’ mango orchard from 59.9% (**Table 1**) to 65%. In both orchards, calcium correction to the desired saturation levels was achieved through the application of *Lithothamnium*-based marine calcium fertilizer (Algen™), which contains 32% Ca²^+^ and 2% Mg^2+^, at doses of 14.60 kg ha⁻¹ for ‘Ataulfo’ and 109.95 kg ha⁻¹ for ‘Kent’.

(ii) After increasing the initial calcium levels in the soil to optimize its condition and promote better plant development, calcium sources were applied to further elevate the Ca²^+^ saturation to 75% in the ‘Ataulfo’ area and to 70% in the ‘Kent’ area. For this purpose, calcium chloride containing 24% Ca²^+^ was applied at a dose of 78 kg ha⁻¹. Calcium complexed with organic acids (Codasal™, composed of 8.7% Ca²^+^; 6.0% N; 14.7% complexing agents in the form of lignosulfonates: Carboxylic acids) and calcium complexed with amino acids (Hendosar™, containing 9.0% N; 6.0% K_2_O; 7.15% Ca; 1.2% Mg and additives with amino acids: L-Threonine ≈3.56%, L-Aspartic Acid -3.45%, L-Serine ≈4.49%, L-Glutamic Acid 9.12%, L-Proline ≈3.50%, L-Glycine -2.43%, L-Alanine ≈2.20%, L-Cystine 2.45%, L-Valine ≈3.10%, L-Methionine -0.23%, L-Isoleucine ≈1.70%, L-Leucine -2.80%, L-Tyrosine ≈1.02%, L-Phenylalanine -1.78%, L-Lysine ≈2.30%, L-Histidine -0.90%, L-Arginine ≈5.20%) were applied at lower doses due to the greater efficiency of these sources, as demonstrated by Tenreiro et al. (2023).

The application schedule for each calcium source was established based on the nutritional requirements of mango. A total of 50% of the recommended dose was applied after pruning, divided into two applications of 25% each, spaced 15 days apart. Subsequently, 20% of the dose was applied at the onset of panicle emergence, and the remaining 30% was distributed during the fruit development stage, split into two applications of 15% each, also spaced 15 days apart.

**Figure 2:**
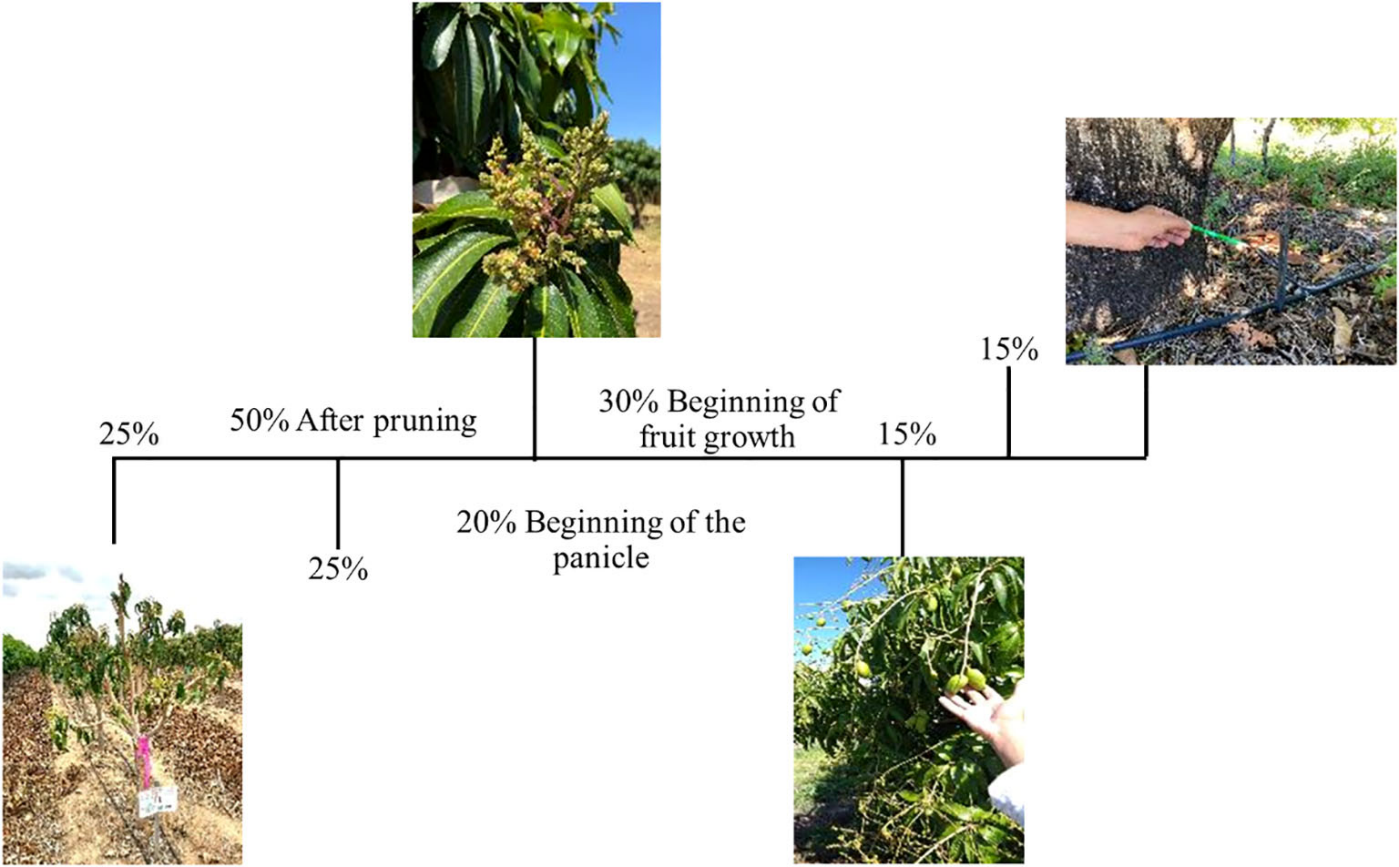
Diagram of calcium source applications (% of the calculated Ca^2+^ dose) and water deficit at 15 days before fruit harvest in experiments with ‘Ataulfo’ and ‘Kent’ mango trees. Source: Adapted from Cavalcante (2023).

The calcium applications were fractionated according to the crop’s calcium demand at each phenological stage, as shown in **Figure 2**, following the criteria proposed by Winston (2007).

Both experiments followed standard agronomic practices for fertilization (except Ca^2+^), crop management, weed control, pest and disease management, and floral induction according to Genú and Pinto (2002), Cavalcante et al. (2018), and Torres (2019). Fifteen days before fruit harvest, irrigation was completely suspended as a water deficit treatment to increase fruit dry mass and improve mango fruit quality (Castro Neto and Reinhardt, 2003).

### 2.3 Evaluated variables

At 284 and 250 days after pruning (DAP), fruits from ‘Ataulfo’ and ‘Kent’ mango trees, respectively, were harvested at ripening stage 2, characterized by a cream-yellow pulp color (Filgueiras, 2000), and weighed to determine fruit yield (kg plant⁻¹). For postharvest analysis, four fruits per plot (16 fruits per treatment) were selected, taken to the laboratory, and stored in a B.O.D. (Biochemical Oxygen Demand) chamber for 15 days at 10°C and subsequently for 7 days at 25°C to simulate maritime transport and shelf-life conditions, respectively (targeting the United States and European Union markets).

Ten fruits per replicate were collected for the determination of N, K^+^, Ca²^+^, and Mg^2+^ concentrations in the pulp, and sent to Plant Soil Laboratories™, Petrolina, Brazil. After washing with distilled water, the pulp was cut, placed in paper bags, and dried in a forced-air oven at 60°C until constant weight. The dried material was ground using a stainless-steel Wiley-type knife mill and stored in hermetically sealed containers. The samples were analyzed for N (mg g⁻¹), K^+^ (mg g⁻¹), Ca²^+^ (mg g⁻¹), and Mg^2+^ (mg g⁻¹) according to the methodology proposed by Tedesco et al. (1995). Additionally, four fruits per treatment were used to form a composite sample for determining cell wall-bound calcium in the pulp, following the methodology described by Bonomelli et al. (2018), with adaptations. The protocol was modified and performed by the private laboratory Plantsoil^®^. To improve sample handling and minimize material loss, the samples were rapidly frozen by immersion in liquid nitrogen and subsequently ground into a fine and homogeneous powder. This approach prevented losses caused by juice leakage and adhesion of residues to container walls, ensuring greater consistency and reliability in the analytical process

The physical and chemical variables evaluated were: fresh fruit mass (FFM), measured with a semi-analytical balance (precision = 0.01 g) and expressed in g; fruit length (FL) and width (FW), measured using a digital caliper (Starret™, 0.01 mm – 300 mm), expressed in mm; pulp firmness (PF), measured using a manual penetrometer with an 8 mm tip at two opposite equatorial positions where the skin was removed, expressed in kgf cm⁻²; pulp dry matter percentage (%DMP), determined by the ratio of fresh pulp mass to dried pulp mass in a forced-air oven at 70°C for 72 hours (until constant weight), multiplied by 100, and expressed as a percentage.

Soluble solids content (SS) was determined by direct reading on an ABBE refractometer and expressed in °Brix; titratable acidity (TA) was measured by titration using 0.1N NaOH and phenolphthalein as an indicator, expressed in g of citric acid per 100 g of fresh pulp; and the SS/TA ratio was calculated as the direct ratio between soluble solids and titratable acidity. Vitamin C (Vit C) was quantified by titration with 2,6-dichlorophenolindophenol (DCPIP), using oxalic acid as the extracting solution. For standardization, 5 mL of the standard ascorbic acid solution was titrated in 50 mL of oxalic acid until a persistent pink coloration was observed for 15 seconds. For the sample, 1 mL of mango pulp juice was diluted in 50 mL of oxalic acid and titrated under the same conditions. All analyses were performed in triplicate (Zenebon et al., 2008).

### 2.4 Statistical analysis

Before the statistical analysis, data were subjected to tests of normality using the Shapiro–Wilk test and homogeneity using the Bartlett test. Subsequently, the data from each experiment, except for the calcium bound to the cell wall of the fruit pulp, were individually analyzed by analysis of variance (ANOVA) using the F-test (p ≤ 0.05). Regarding calcium bound to the pulp cell wall, only descriptive analysis of the data was performed. The means related to irrigation levels and calcium sources were compared using Tukey’s test at a 5% significance level. Statistical analyses were performed using Sisvar software version 5.6 (Ferreira, 2019).

Multivariate analysis (MANOVA) was also performed using the bidimensional hierarchical clustering technique with graphical representation in the form of a heatmap. Data were previously normalized using z-score standardization and arranged in matrices containing the evaluated variables and treatments. Euclidean distances were calculated to assess the similarity between variables and treatments, using the complete linkage method to construct the dendrograms. Visualization was carried out using the *heatmap.2* function from the *gplots* package in R environment, allowing the identification of correlation patterns, clusters, and contrasts among the different traits of post-harvest quality and fruit production components.

## 3 Results

**Figure 3:**
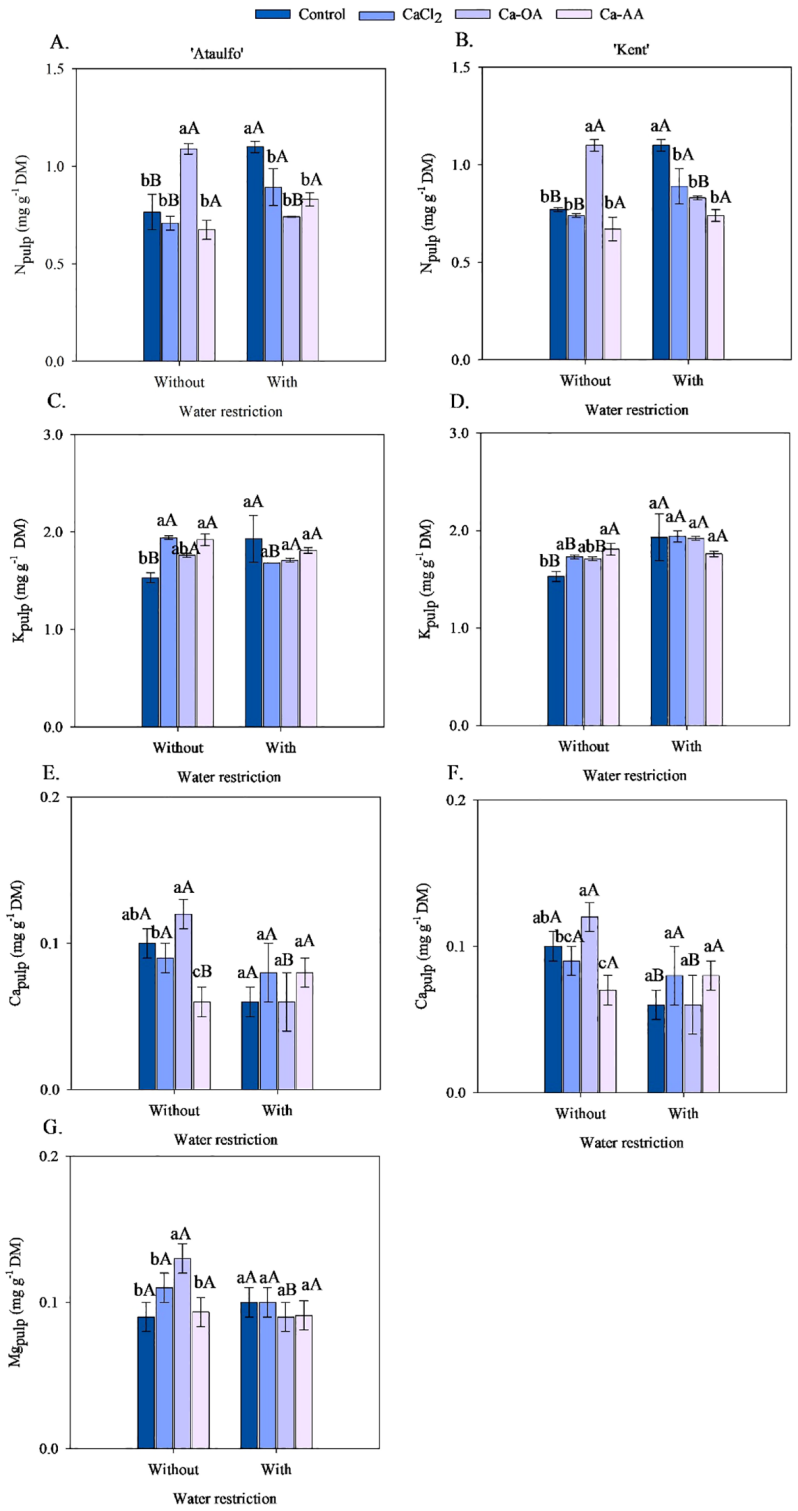
Mineral composition of the pulp of ‘Ataulfo’ **(A, C, E, G)** and ‘Kent’ **(B, D, F)** mangoes as affected by calcium fertilization and water deficit. CaCl_2_ – Calcium chloride; Ca-OA – Calcium complexed with organic acids; Ca-AA – Calcium complexed with amino acids. Bars with the same lowercase letter do not differ from each other with respect to calcium source for same water restriction condition according to Tukey’s test at 5% probability. Bars with the same uppercase letter do not differ from each other for same calcium source under different water restriction condition according to Tukey’s test at 5% probability. The dispersion above the bars represents the standard deviation of the mean (n = 4).

The mineral composition of nitrogen (N), potassium (K^+^), calcium (Ca^2+^), and magnesium (Mg^2+^) in the pulp of ‘Ataulfo’ and ‘Kent’ mangoes, as affected by calcium fertilization and pre-harvest water restriction, is presented in **Figure 3**. Nitrogen concentrations in the fruit pulp displayed a similar distribution across both cultivars and water regimes (**Figures 3A, B**). In plants not subjected to water restriction, the application of calcium complexed with organic acids resulted in the highest N concentrations in the pulp, with increases of 43.17% for ‘Ataulfo’ and 43.16% for ‘Kent’ compared to the control. Notably, under water restriction, N levels in the pulp increased in both cultivars when no calcium was applied.

Pre-harvest water restriction increased N concentration in the pulp of plants without calcium and those treated with CaCl_2_, with gains of 44.15% and 25.94% for ‘Ataulfo’, and 44.14% and 19.99% for ‘Kent’, respectively (**Figures 3A, B**). In treatments without water deficit, Ca-OA fertilization raised N content from 0.702 to 1.096 mg g^-1^ DM in ‘Ataulfo’ and from 0.831 to 1.096 mg g^-1^ DM in ‘Kent’. No effect of water restriction was observed on N levels in Ca-AA treatments.

For K^+^ concentrations in the pulp (**Figures 3C, D**), there is interaction between calcium sources and water deficit was detected. However, under non-restricted irrigation, CaCl_2_ and Ca-AA enhanced K^+^ levels in the pulp, with increases of 26.51% and 25.60% in ‘Ataulfo’, and 13.33% and 18.48% in ‘Kent’ compared to the control. In ‘Ataulfo’, water restriction raised K^+^ levels only when calcium was not applied, while a reduction was observed with CaCl_2_. In ‘Kent’, water deficit increased K^+^ content in all treatments except Ca-AA.

The application of Ca-OA under water restriction elevated Ca^2+^ levels in the pulp of both cultivars by approximately 22.3% relative to the control (**Figures 3E, F**). The highest pulp Ca^2+^ concentrations under water restriction were obtained with CaCl_2_ and Ca-AA. In ‘Ataulfo’, water restriction decreased pulp Ca^2+^ levels with Ca-OA (from 0.117 to 0.063 mg g^-1^ DM), with no significant differences for other sources. In ‘Kent’, Ca levels declined under water restriction in the control and Ca-OA treatments by 66.67% and 85.87%, respectively.

Water restriction before harvest reduced the Ca^2+^ pulp concentration in ‘Ataulfo’ fruits from 0.117 to 0.063 mg g^-1^ DM when combined with the application of Ca-OA, while no difference was observed for the other calcium sources (**Figure 3E**). In the fruits of ‘Kent’, the Ca^2+^ pulp concentration was reduced with water restriction in the treatments without calcium and Ca-OA, respectively, by 66.67% and 85.87% (**Figure 3F**).

For magnesium, a significant interaction between calcium source and water restriction was observed only in ‘Ataulfo’ (**Figure 3G**). Ca-OA promoted the highest Mg concentration in the pulp, increasing it by 42.22% compared to the control. Under water deficit, no statistical differences were detected among sources, except that Ca-OA combined with water restriction reduced Mg from 0.131 to 0.089 mg g^-1^ DM.

**Figure 4:**
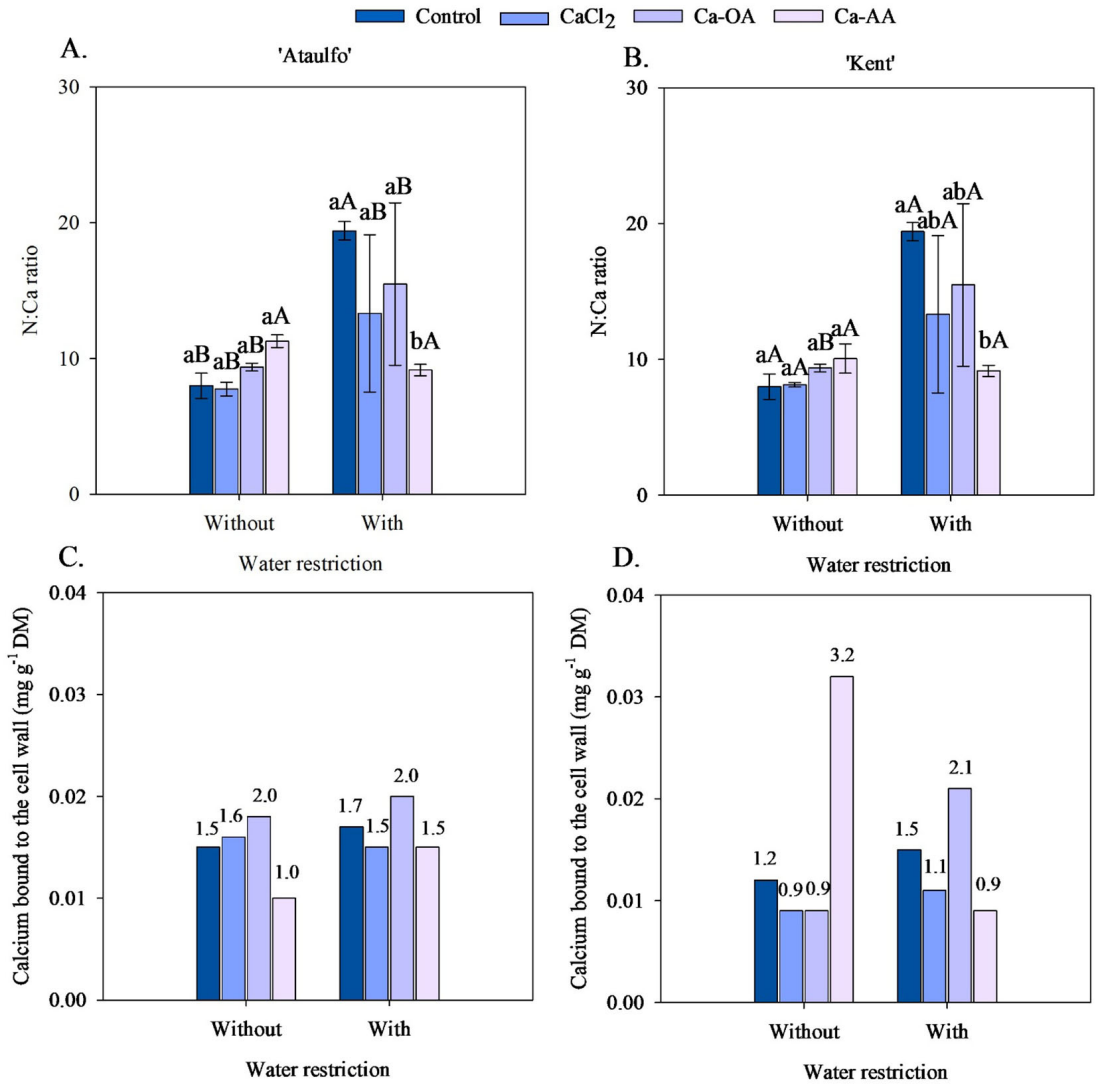
N:Ca ratio and cell wall-bound calcium concentration in the pulp of ‘Ataulfo’ **(A, C)** and ‘Kent’ **(B, D)** mangoes as affected by calcium fertilization and water deficit. Control – no calcium; CaCl_2_ – Calcium chloride; Ca-OA – Calcium complexed with organic acids; Ca-AA – Calcium complexed with amino acids. Bars with the same lowercase letter do not differ from each other with respect to calcium source for same water restriction condition according to Tukey’s test at 5% probability. Bars with the same uppercase letter do not differ from each other for the same calcium source under different water restriction conditions according to Tukey’s test at 5% probability. The dispersion above the bars **(A, B)** represents the standard deviation from the mean (n = 4).

The N:Ca ratio in the pulp of ‘Ataulfo’ and ‘Kent’ mango fruits without pre-harvest water restriction did not show statistical differences with the application of calcium sources (**Figures 4A, B**). However, under water restriction, the N:Ca ratio in the pulp of the mango cultivars increased when no calcium was applied, although it did not differ statistically from the treatments with CaCl_2_ and Ca-OA. The application of calcium complexed with amino acids reduced the N:Ca ratio in the fruits of both cultivars, with a reduction of 52.84% (‘Ataulfo’) and 52.85% (‘Kent’) compared to the control treatment.

Except in the treatments with the application of Ca-AA, water restriction increased the N:Ca ratio in the pulp of mango fruits (**Figures 3A, B**). Water restriction before harvest increased the N:Ca ratio in ‘Ataulfo’ and ‘Kent’, respectively, by 142.94 and 142.89% (control), 71.89 and 63.84% (CaCl_2_), and 65.40 and 65.63% (Ca-OA).

The highest values of cell wall-bound calcium concentration in fruits of ‘Ataulfo’ mangoes were observed in the treatments with the application of calcium complexed with organic acids, regardless of the application of water restriction before harvest (**Figure 4C**), with values of 0.018 mg.g^-1^ DM (without restriction) and 0.020 mg.g^-1^ DM (with restriction). In the ‘Kent’ cultivar, it was observed that the application of complexed sources promoted higher values of cell wall-bound calcium concentration of fruits, but the source varied depending on the application or not of the water restriction before harvest (**Figure 4D**). The highest cell wall-bound calcium concentration was observed when Ca-AA was applied to plants that did not receive water restriction, with a value of 0.032 mg.g^-1^ DM; while, in the water restriction treatment, the application of Ca-OA promoted the highest cell wall-bound calcium concentration in fruits (0.021 mg g^-1^ DM).

**Figure 5:**
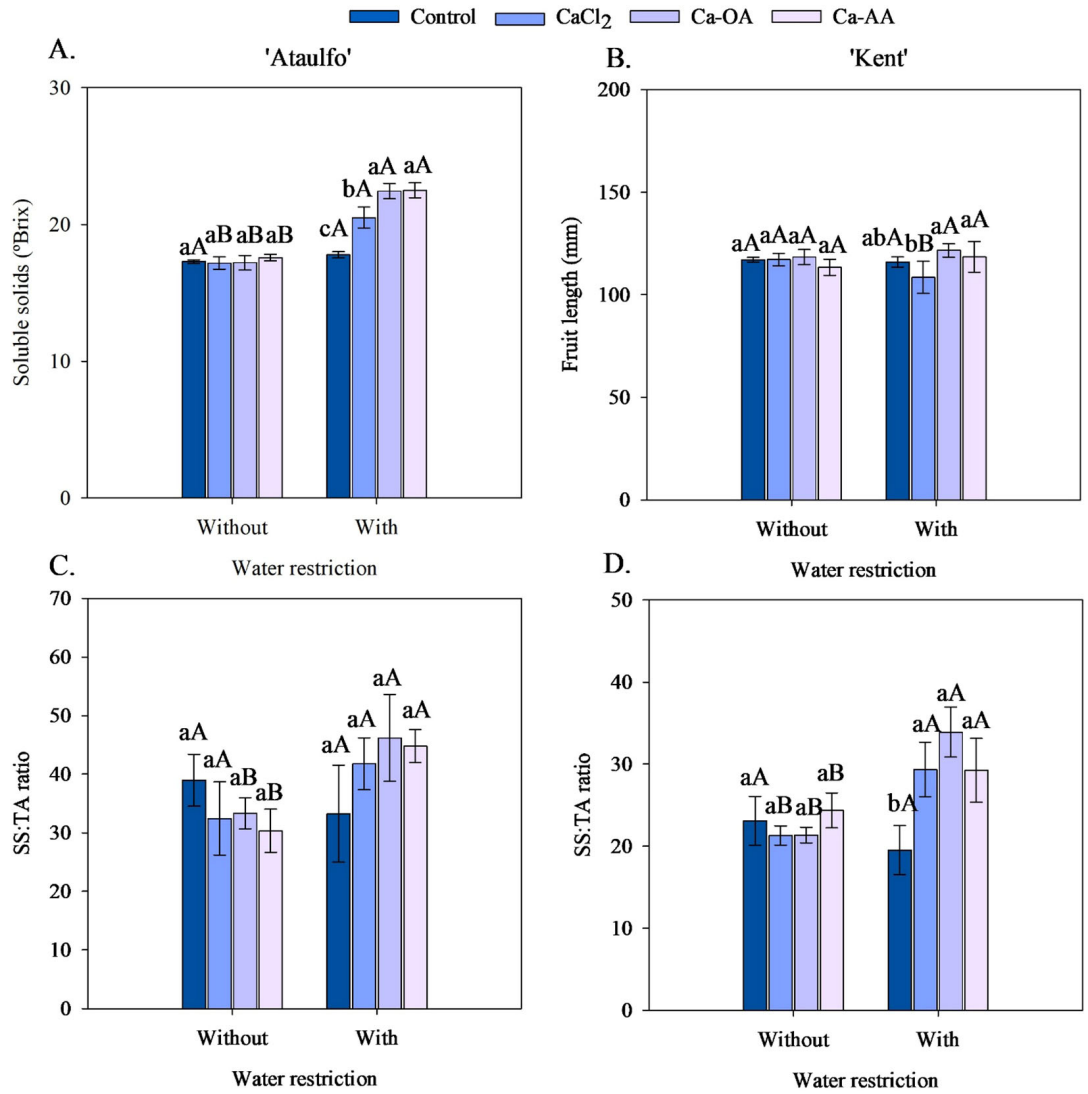
Soluble solids content **(A)** and SS: TA ratio **(C)** of ‘Ataulfo’ mango, and fruit length **(B)** and SS: TA ratio **(D)** of ‘Kent’ mango as affected by calcium fertilization and water deficit. Control – no calcium; CaCl_2_ – Calcium chloride; Ca-OA – Calcium complexed with organic acids; Ca-AA – Calcium complexed with amino acids. Bars with the same lowercase letter do not differ from each other with respect to calcium source for same water restriction condition according to Tukey’s test at 5% probability. Bars with the same uppercase letter do not differ from each other for the same calcium source under different water restriction conditions according to Tukey’s test at 5% probability. The dispersion above the bars represents the standard deviation from the mean (n = 4).

The application of calcium sources without water restriction did not affect the soluble solids in the pulp of ‘Ataulfo’ mango fruits (**Figure 5A**). However, under water restriction, it was observed that the application of complexed calcium sources increased the soluble solids in the fruits, with increments of 26.20% and 26.54% with the application of Ca-OA and Ca-AA compared to the fruits of the control treatment. Except for the treatment without calcium, the soluble solids content was increased by 19.32% (CaCl_2_), 30.38% (Ca-OA), and 27.98% (Ca-AA) with the application of water restriction before harvest. No effects of the treatments on soluble solids and titratable acidity were recorded for ‘Kent’.

As shown in **Figure 5B**, the length of ‘Kent’ mango fruits was not affected by the calcium sources without water restriction. However, the application of complexed sources increased the fruit length of the ‘Kent’ from 116.00 cm (control) to 121.68 cm (Ca-OA) and 118.47 cm (Ca-AA) when associated with water restriction. Water restriction reduced the fruit length of the ‘Kent’ cultivar by 7.96% in the treatment with CaCl_2_ application, but did not affect the variable in the other calcium sources.

The calcium sources did not promote significant effects on the SS: TA ratio in the fruits of ‘Ataulfo’ and ‘Kent’ mangoes in the treatments without water restriction before harvest (**Figures 5C, D**). However, the fruits of ‘Kent’ mangoes had a higher SS: TA ratio when Ca-OA or Ca-AA was applied compared to the control (**Figure 5D**). Comparatively, CaCl_2_, Ca-OA, and Ca-AA promoted increments in this variable of 25.65%, 38.91%, and 34.70%, respectively. In the ‘Ataulfo’, the calcium sources did not affect the SS: TA ratio in the fruits of the treatment with water restriction before harvest (**Figure 5C**).

As shown in **Figure 5C**, water restriction increased the SS: TA ratio in ‘Ataulfo’ mango fruits when associated with the application of the complexed calcium sources, from 33.20 to 46.19 (Ca-OA) and from 30.33 to 44.79 (Ca-AA). Similarly, the SS: TA ratio of ‘Kent’ mango fruits was elevated with water restriction in all treatments where calcium was applied to the plants, especially in the complexed organic acid source (Ca-OA), which showed increments of 38.63% (**Figure 5D**).

**Figure 6:**
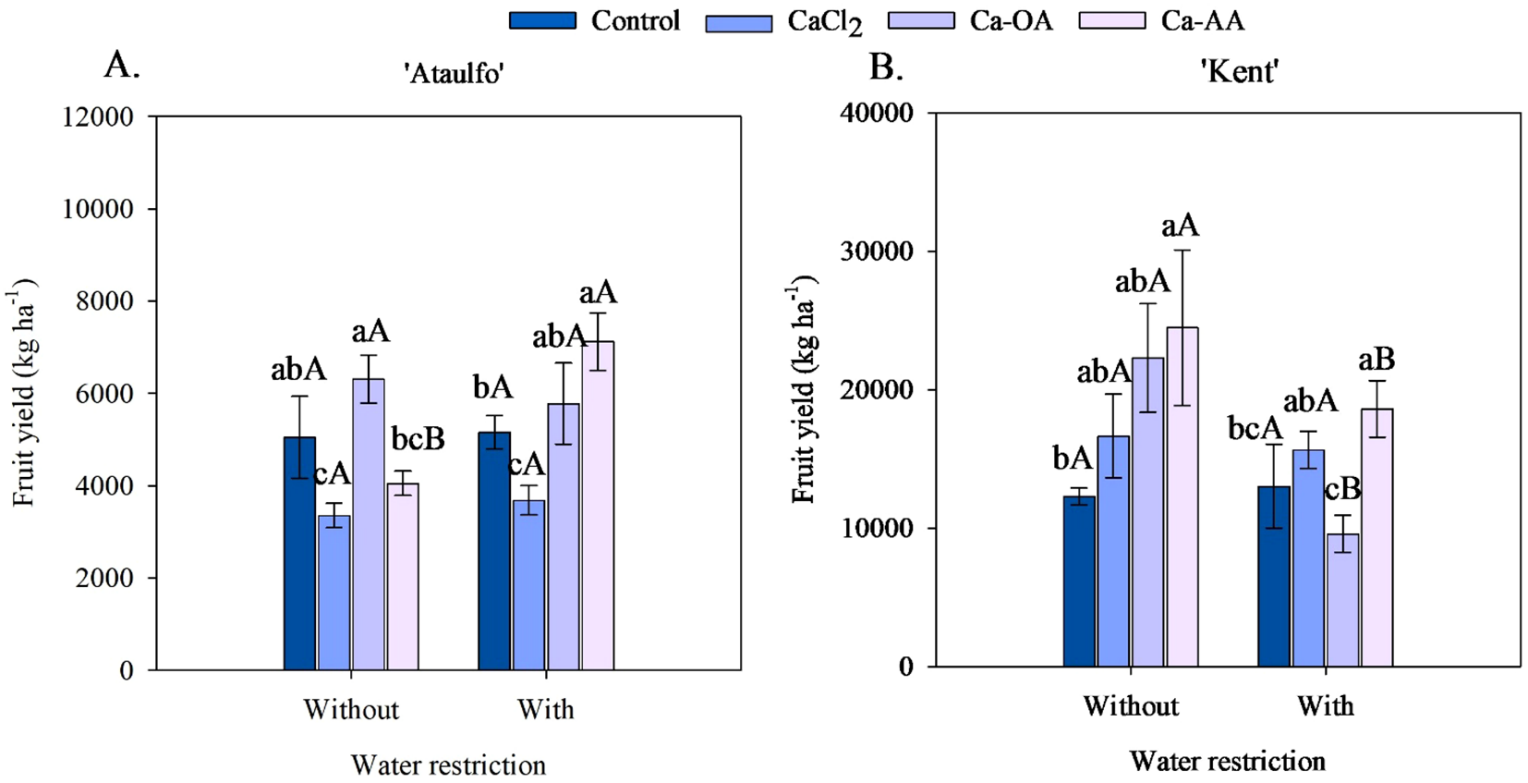
Fruit yield of ‘Ataulfo’ **(A)** and ‘Kent’ **(B)** mangoes as affected by calcium fertilization and water deficit. Control – no calcium; CaCl_2_ – Calcium chloride; Ca-OA – Calcium complexed with organic acids; Ca-AA – Calcium complexed with amino acids. Bars with the same lowercase letter do not differ from each other with respect to calcium source for same water restriction condition according to Tukey’s test at 5% probability. Bars with the same uppercase letter do not differ from each other for same calcium source under different water restriction condition according to Tukey’s test at 5% probability. The dispersion above the bars represents the standard deviation of the mean (n = 4).

The application of complexed calcium sources increased the fruit yield of ‘Ataulfo’ and ‘Kent’ mangoes, regardless of water restriction before harvest (**Figure 6**). Under water restriction, the highest fruit yield in ‘Ataulfo’ mangoes was in the treatment with Ca-OA application (6,309.79 kg ha^-1^), while for ‘Kent’, it was with Ca-AA application (24,468.67 kg ha^-1^), as shown in **Figures 6A, B**. Compared to the control treatment (no calcium), these values were increased by 24.91% and 99.02%, respectively, in ‘Ataulfo’ and ‘Kent’ mangoes. The Ca-AA increased the fruit yield of both ‘Ataulfo’ and ‘Kent’ mangoes under water restriction before harvest (**Figure 6**). Ca-AA increased the fruit yield in ‘Ataulfo’ by 38.03% (**Figure 6A**) and in ‘Kent’ by 42.79% (**Figure 6B**).

Water restriction before harvest had a distinct effect on the fruit yield of the mango cultivars (**Figure 6**). In ‘Ataulfo’ mango, water restriction caused a significant effect on plants fertilized with Ca-AA, increasing the fruit yield from 4054.12 to 7123.21 kg ha^-1^, while in the other treatments, no effect of water restriction was observed (**Figure 6A**). In contrast, in ‘Kent’ mango, the fruit yields of plants with Ca-OA and Ca-AA applications were reduced by 57% and 24%, respectively, with the application of water restriction (**Figure 6B**).

**Figure 7:**
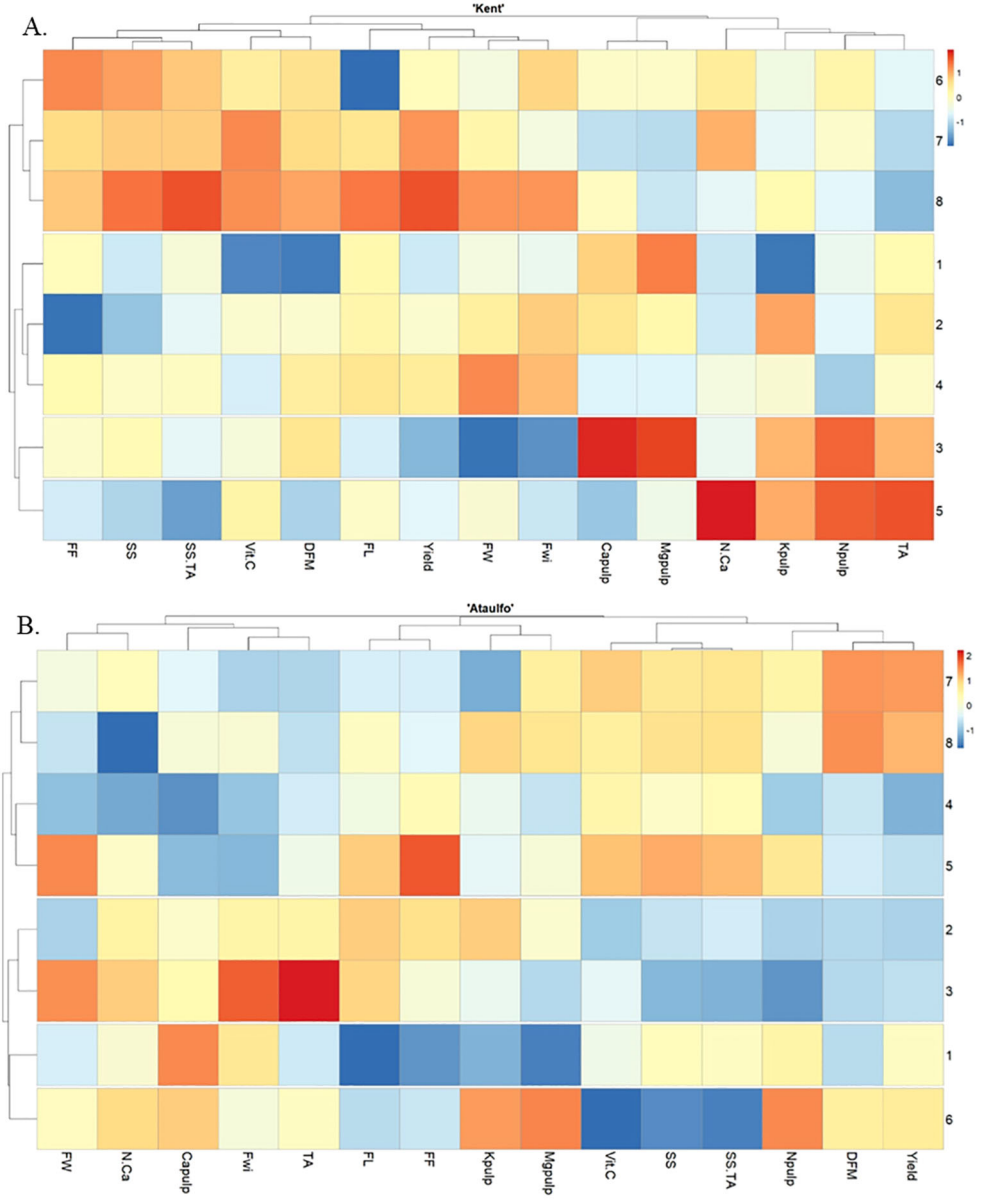
Heatmap representation of the hierarchical clustering analysis based on Pearson correlation of ‘Kent’ **(A)** and ‘Ataulfo’**(B)** mango samples as affected by calcium fertilization and water deficit. TA, Titratable acidity; Npulp, Nitrogen in the pulp; Kpulp, Potassium in the pulp; N.Ca, Nitrogen-to-calcium ratio in the pulp; Mgpulp, Magnesium in the pulp; Capulp, Calcium in the pulp; Fwi, Fruit width; FW, Fruit weight; Yield, Fruit yield; FL, Fruit length; DFM, Fruit dry matter; Vit.C, Vitamin C; SS.TA, Soluble solids-to-titratable acidity ratio; SS, Soluble solids; FF, Fruit firmness.

The heatmap presented illustrates the variation of different parameters in relation to the applied treatments for ‘Kent’ and ‘Ataulfo’ mangoes (**Figures 7A, B**, respectively), where the treatments vary with the presence or absence of calcium and with or without water restriction. The dendrogram analysis suggests the proximity and similarity of the parameters and treatments, facilitating the visualization of clusters, and the intensity of the colors indicates the magnitude and direction of the interactions (blue indicating negative correlations, orange/red indicating positive correlations).

Clear differences can be observed between conditions with and without water restriction by examining lines 1 to 4 in the heatmap for ‘Kent’, which represent treatments without water restriction, while lines 5 to 8 correspond to the same treatments with water restriction. The differences in color gradients (blue and orange/red) indicate significant changes in parameters, particularly in the variables N.Ca and Capulp, where the presence of water restriction (lines 5 to 8) caused a stronger distinction (with intense red colors for higher response levels). This suggests that, under water restriction, the accumulation of calcium and pulp yield were significantly altered.

In the treatments without water restriction (lines 1 to 4), there appears to be less variation in the analyzed parameters, except for a few responses, such as the variables SS and FF, which show slight differentiation. The application of CaCl_2_ (treatment 2) without water restriction did not cause a significant variation compared to the control (treatment 1), suggesting a lesser influence on this set of variables.

To address the effect of water restriction in calcium treatments (lines 5-8) in ‘Kent’, it is important to highlight that the impact of water restriction on the parameters is clearly visible, especially in the variables “Capulp”, “N.Ca”, “MgPulp”, and “Yield”, where warm colors (orange/red) dominate the restricted treatments (lines 5-8), indicating an intense response to this condition. This suggests that, under water restriction, the accumulation of nutrients like calcium and magnesium in the pulp of ‘Kent’ mangoes was more pronounced, especially in the treatment Ca-OA.

The dendrogram also highlights how the parameters are correlated. For example, fruit quality-related variables, such as “Yield”, “FW”, “Fwi”, and “Capulp”, form a group, suggesting a correlation between these variables in response to the treatments. Similarly, “N.Ca”, “Kpulp”, and “Npulp” are grouped together, indicating a potential interaction between these nutrients and the effect of calcium treatments.

Thus, the heatmap reveals that water restriction had a significant impact on the analyzed parameters, especially on nutrient accumulation in the pulp and fruit yield. Moreover, calcium applications, particularly Ca-OA and Ca-AA, seem to have modulated this response, with differentiated effects between conditions with and without water restriction for ‘Kent’ mango.

For ‘Ataulfo’ mango without water restriction (line 1), a positive correlation with fruit firmness (FW) and antioxidant capacity (Vit.C) is observed, suggesting that, in the absence of calcium, the fruit has lower firmness and antioxidant activity. With water restriction (line 5), there is a general increase in correlations with firmness and antioxidants, indicating that water restriction may enhance these parameters in the absence of calcium.

For the treatment without water restriction (line 2), CaCl_2_ appears to negatively impact firmness (FW) and the soluble solids/titratable acidity ratio (SS.TA), suggesting a decrease in pulp quality with this treatment. Furthermore, with water restriction (line 6), a negative correlation with vitamin C (Vit. C) is observed, indicating that water restriction combined with CaCl_2_ may reduce ascorbic acid content.

The treatment containing Ca-OA without water restriction (line 3) shows a strong positive correlation with firmness (FW) and a negative correlation with fruit yield (Yield), suggesting an interesting impact on production compared to the same treatment with Ca-OA under water restriction (line 7), where the positive correlation with fruit yield (Yield) remains, but there is a negative impact on other parameters, such as firmness.

The treatment with Ca-OA without water restriction (line 4) for ‘Ataulfo’ mango seems to have varied effects, with a strong negative correlation with titratable acidity (TA), indicating that this combination hinders the increase in acidity. In contrast, with water restriction (line 8), there is a strong positive correlation with DFM and fruit yield (Yield), suggesting that the combination of ascorbic acid with water stress may increase physical resistance and fruit yield in ‘Ataulfo’ mangoes.

Water restriction, combined with calcium, seems to improve some nutritional parameters, such as vitamin C (with CaCl_2_) and firmness. This indicates that water stress can be a strategy to improve certain quality attributes, provided it is paired with an adequate calcium source. The different types of calcium influence fruit quality in varied ways: Ca-AA has negative impacts on firmness but positive effects on fruit yield, while vitamin C has interesting effects on firmness and acidity, especially under water stress.

## 4 Discussion

The results presented in the study demonstrate a complex interaction between the suspension of the water fifteen days before harvest (with and without water restriction) and the calcium sources applied (control – no calcium; calcium chloride – CaCl_2_, calcium complexed with organic acids – Ca-OA, and calcium complexed with amino acids – Ca-AA). This interaction affects biochemical and agronomic parameters in both mango cultivars (‘Ataulfo’ and ‘Kent’), revealing significant variations in mineral content, fruit quality, and plant yield. The main results of the study for each evaluated cultivar will be discussed below.

The application of complexed calcium (Ca-OA and Ca-AA) increased N pulp concentrations, especially under conditions without water restriction. This result can be explained by the effect of water stress on the concentration of solutes in the pulp, reducing N dilution in the liquid fraction of the fruit (Conde et al., 2015). Furthermore, the higher calcium availability plays a crucial role in nutrient absorption and ion transport via the xylem (Saito and Uozumi, 2020).

Water deficit increased N pulp concentrations in plants without calcium or treated with CaCl_2_, indicating that the response to water stress involves physiological mechanisms that may alter nutrient mobilization. However, plants treated with Ca-AA were not influenced by water restriction, suggesting that its complexed form of calcium provides greater nutritional stability. In non-water-stressed treatments, Ca-OA promoted the highest nitrogen levels, indicating a positive effect of Ca-OA on nitrogen absorption, possibly due to the greater transport efficiency of complexed calcium, which can stimulate nitrate assimilation through transporter regulation (Marschner, 2023).

Potassium pulp concentrations were more affected by water restriction than by the calcium sources. In the absence of calcium (control treatment), water restriction increased potassium levels, possibly due to the redistribution of ions to maintain osmotic balance during stress and cellular turgor. In this scenario, the absence of calcium accentuated the effect, as calcium helps stabilize membranes and cellular structures (Torres et al., 2024). In this sense, the low availability of calcium in soil that did not receive calcium via fertilization may have affected the K:Ca balance in the soil. The increase in K^+^ ions in the solution, coupled with the low availability of Ca^2+^ ions, may have reduced calcium absorption due to an antagonistic relationship between the elements, especially since, in non-selective cation channels (NSCCs), the concentration of the cation in the solution is the predominant factor for greater or lesser absorption in relation to another (Hernández-Peréz et al., 2020). In addition, without calcium, membranes become more permeable, facilitating potassium accumulation (Shabala et al., 2006; Ebeed et al., 2024). Thus, the increase in potassium may be a plant response to compensate for the lack of calcium and minimize drought-induced damage, maintaining homeostasis and cellular function.

Calcium sources such as CaCl_2_ and Ca-AA promoted significant increases in potassium levels under adequate water supply conditions. However, under water restriction, this effect was reduced. This response may be related to the known antagonistic interaction between potassium and calcium during root uptake, as widely described in the literature (Elephant et al., 2023; Qu et al., 2023). It is important to note that competition between Ca²^+^ and K^+^ can occur under both adequate water supply and water-deficit conditions, but the extent of this interaction may be more pronounced during water stress due to altered root physiology and ion transport dynamics. This could explain the reduced potassium accumulation under water deficit despite calcium application.

In the ‘Kent’ cultivar, water restriction increased K^+^ pulp levels in fruits from plants that did not receive calcium, while the other treatments remained constant (**Figure 3D**). This increase may be associated with the activation of potassium channels in response to water stress, a mechanism already observed in crops such as grapes - *Vitis vinifera* (Liu et al., 2023), but not yet mentioned in the scientific literature for mango.

The complexed calcium source Ca-OA was more effective in increasing Ca^2+^ pulp concentrations when water restriction was applied, regardless of the mango cultivar evaluated (**Figures 3E, F**). This result can be attributed to the likely greater mobility and availability of complexed calcium, which favors its absorption by roots and translocation to storage tissues, such as the fruits, as pointed out by Wójcik and Wójcik (2003). Complexation with organic acids enhances nutrient solubility and reduces its fixation in the soil (Madhupriyaa et al., 2024), which may explain the increase in calcium levels detected in the pulp under ideal water conditions.

Under water stress, however, the differences between calcium sources were attenuated, indicating a limitation in nutrient absorption and transport. This likely occurred because calcium is predominantly transported via the xylem, whose efficiency depends on transpiration flow (Kabir and Díaz-Pérez, 2025). In drought conditions, reduced transpiration compromises calcium movement to the fruits, making the effects of different formulations less pronounced. Previous studies also highlight that water stress can alter the distribution and allocation of nutrients, hindering calcium accumulation in low-transpiration organs, such as fruits (Sena et al., 2024; Torres et al., 2024).

Regarding Mg^2+^ pulp concentrations (**Figure 3G**), the interaction between the studied factors was only observed for the ‘Ataulfo’ cultivar. Ca-OA promoted the highest Mg pulp concentrations when no water restriction was applied, while water restriction reduced the Mg^2+^ levels when combined with Ca-OA. This result can be explained by the action of organic acids as chelating agents, which favor the solubilization and mobility of cations in the soil, including Mg²^+^ (Mengel and Kirkby, 2001; Ram et al., 2024).

Moreover, higher root activity under ideal water conditions may have contributed to the joint absorption of Ca^2+^ and Mg^2+^ (Gomes et al., 2024), promoting nutrient accumulation in the fruits. On the other hand, when water deficit was applied, Mg^2+^ pulp concentrations under treatment Ca-OA were reduced, suggesting possible antagonism in cation absorption under stress, as this condition results in lower mass flow in the soil solution and reduced transpiration, hindering the transport of mobile nutrients such as Mg²^+^ (Qu and Han, 2022). Additionally, excessive calcium may compete with magnesium for absorption sites in the root, exacerbating in soils with low water availability (Saito and Uozumi, 2020). Thus, the data suggest that although Ca-OA enhances Mg^2+^ absorption under ideal conditions, its combination with water stress may lead to nutritional imbalances that negatively affect magnesium accumulation in fruits.

Water restriction increased the N:Ca ratio in pulp by 142.94% (‘Ataulfo’) and 142.89% (‘Kent’) in the control treatment. This increase is concerning, as a high N:Ca ratio is associated with physiological disorders, although none of the treatments studied reached the N:Ca ratio of 31.0, which Assis et al. (2004) stated is critical for the occurrence of ‘Jelly seed’ in mangoes. On the other hand, the control treatment in both situations provided values above the N:Ca ratio in pulp of 17.13 (Assis et al., 2004).

The treatment with Ca-AA was the only one to reduce the N:Ca ratio under water restriction, with decreases of 52.84% (‘Ataulfo’) and 52.85% (‘Kent’), suggesting that this form of calcium may be more efficient in regulating the N:Ca balance, possibly by promoting calcium assimilation and redistribution relative to nitrogen, even under adverse conditions. The complexation of calcium with amino acids may facilitate its transport via the phloem, as well as improve its root absorption through active transport mechanisms mediated by peptides, contributing to a better ionic balance between nitrogen and calcium in the cells, as reported by Epstein and Bloom (2005) and Bellandi et al. (2022).

As shown in **Figures 4C, D**, there was an increase in calcium bound to the cell wall with the use of Ca-AA, especially in the ‘Kent’ cultivar under water restriction, reinforcing the hypothesis of greater physiological efficiency of this formulation. According to Marschner (2023), complexed calcium may be less susceptible to precipitation in the soil and more efficient in providing the nutrient sustainably, which is particularly advantageous under stress conditions where cation absorption may be compromised.

Soluble solids (°Brix) (**Figure 5A**) increased under water restriction with the application of calcium sources, possibly due to the water-concentrating effect in the tissues, as well as the potential role of calcium in preserving cell integrity and carbohydrate metabolism (Giorgi et al., 2018). According to Feng et al. (2023), calcium acts on membrane stability and may contribute to preserving soluble sugars under adverse conditions. In addition, Taiz et al. (2017) affirm that solute accumulation in response to water stress is a common tolerance mechanism in fruit trees, favoring osmotic adjustment and maintaining basic metabolism.

In the ‘Kent’ cultivar, water restriction reduced fruit length in plants treated with CaCl_2_ by 7.96% (**Figure 5B**), but did not affect other treatments, suggesting that CaCl_2_ may be less effective in maintaining growth under water stress.

The SS: TA ratio, an important sensory quality indicator, was positively impacted by water restriction and by complexed calcium sources (Khakpour et al., 2022). In ‘Kent’ fruits, Ca-OA promoted the highest increases (**Figure 5**), suggesting that this type of fertilizer can modulate acidity and sugar content in a beneficial way. Calcium may slow down the maturation metabolism, reduce respiration, and preserve soluble solids and acidity levels, favoring fruit flavor and acceptance (Epstein and Bloom, 2005; Alves et al., 2023).

The application of Ca^2+^ sources positively influenced the fruit yield of ‘Ataulfo’ and ‘Kent’ cultivars, with variations depending on water availability (**Figures 6A, B**). The best performance for ‘Ataulfo’ was recorded with the use of Ca-OA, especially in the absence of water restriction, reinforcing the role of this formulation in improving nutrient absorption and translocation efficiency. Complexes with organic acids promote greater calcium solubility and mobility, enhancing its absorption by roots even in environments with higher ionic competition (Ram et al., 2024). In this same cultivar, there was an increase in plant yield with Ca-AA treatment, from 4,054.12 to 7,123.21 kg ha^-1^, further reinforcing that this source is beneficial under water stress and had the highest average fruit yield for ‘Ataulfo’.

For ‘Kent’, the best results were recorded when Ca-AA was adopted under both non-restricted and water-restricted conditions (**Figure 6B**). Especially in ‘Kent’ mango under water restriction, although fruit yield decreased in all treatments, CaCl_2_ and Ca-AA sources helped partially mitigate the negative effects of water stress.

These results align with the fact that calcium participates in signaling mechanisms and maintains cell membrane integrity, being crucial in adaptive responses to water deficit (Verna et al., 2022; Naz et al., 2024). Thus, adequate calcium management contributes to improving plant resistance to water stress, positively influencing fruit yield and fruit quality.

## 5 Conclusions

This study demonstrates that the combination of water restriction and complexed calcium sources can be a promising strategy to optimize the quality and fruit yield of mango trees. However, the effects are highly dependent on the cultivar and the form of calcium used.

The application of calcium sources, especially in complexed forms with organic acids (Ca-OA) and amino acids (Ca-AA), improves the mineral composition, quality, and fruit yield of ‘Ataulfo’ and ‘Kent’ mangoes, with responses varying according to cultivar and the water restriction.

Ca-OA is more effective under full irrigation, increasing N, Ca^2+^, and Mg^2+^ levels in the pulp, while Ca-AA stands out under water restriction by reducing the N:Ca ratio and increasing calcium bound to the cell wall.

For ‘Ataulfo’, it is recommended to apply water deficit before harvest and, throughout the productive cycle use Ca-AA. For ‘Kent’, suspension of irrigation during the pre-harvest period is not recommended, with Ca-AA also being the preferred calcium source.

## Data Availability

The original contributions presented in the study are included in the article/**Supplementary Material**. Further inquiries can be directed to the corresponding author.
