# Supplementary figures and images for: Combined effects of calcium sources and water restriction on fruit yield and quality of ‘Ataulfo’ and ‘Kent’ mangoes
Source: Front Plant Sci. 2025 Aug 28;16:1622533. doi: 10.3389/fpls.2025.1622533 (PMC12423558; doi:10.3389/fpls.2025.1622533)

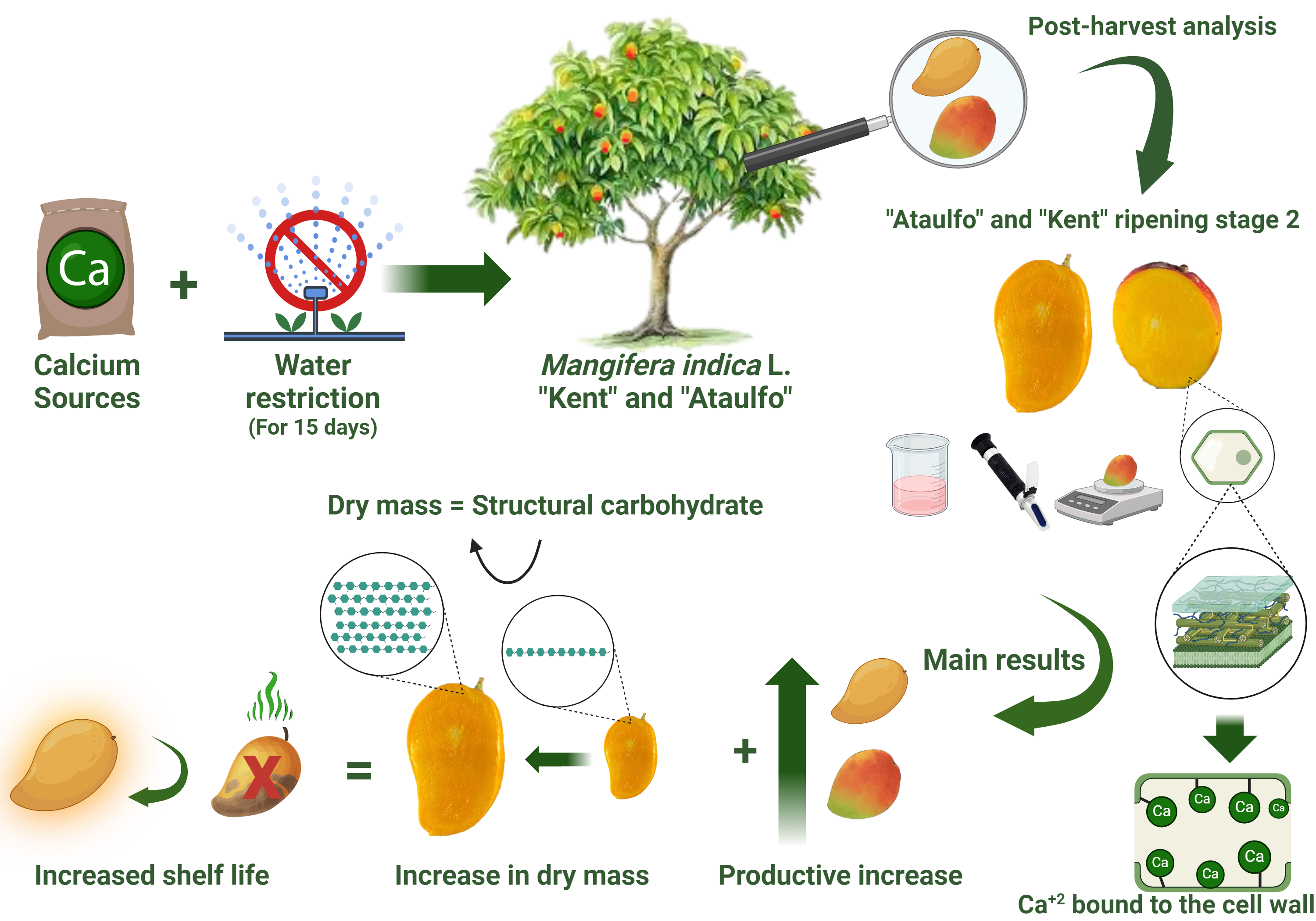

Supplement: Supplementary file 1 [file Image1.png]
